# Children’s Use of Communicative Intent in the Selection of Cooperative Partners

**DOI:** 10.1371/journal.pone.0061804

**Published:** 2013-04-23

**Authors:** Kristen A. Dunfield, Valerie A. Kuhlmeier, Lindsay Murphy

**Affiliations:** 1 The Department of Psychology, Concordia University, Montreal, Quebec, Canada; 2 The Department of Psychology, Queen’s University, Kingston, Ontario, Canada; Hungarian Academy of Sciences, Hungary

## Abstract

Within the animal kingdom, human cooperation represents an outlier. As such, there has been great interest across a number of fields in identifying the factors that support the complex and flexible variety of cooperation that is uniquely human. The ability to identify and preferentially interact with better social partners (partner choice) is proposed to be a major factor in maintaining costly cooperation between individuals. Here we show that the ability to engage in flexible and effective partner choice behavior can be traced back to early childhood. Specifically, across two studies, we demonstrate that by 3 years of age, children identify effective communication as “helpful” (Experiments 1 & 2), reward good communicators with information (Experiment 1), and selectively reciprocate communication with diverse cooperative acts (Experiment 2). Taken together, these results suggest that even in early childhood, humans take advantage of cooperative benefits, while mitigating free-rider risks, through appropriate partner choice behavior.

## Introduction

Humans are an extraordinarily cooperative species. Yet, unless exercised with caution, the tendency to act on behalf of others is risky. Specifically, individuals who are indiscriminately cooperative can be exploited by ‘free riders’, those who reap the benefits of cooperation without ever bearing the costs, thus undermining the success of cooperative individuals [1]. This observation has led to great interest, across many fields of inquiry, in identifying the factors that support human cooperation. Indeed, an impressive body of research in the fields of behavioral and biological sciences suggests that individuals have many ways to exploit the benefits of cooperation while mitigating the risks. Specifically, mechanisms for maintaining cooperation between unrelated individuals can be divided into at least two categories of evolutionarily stable strategies that support reciprocity, one set involving partner fidelity (partner control) and another involving partner choice [2], [3].

In partner control models, the same two individuals engage in an iterated, indefinite, series of interactions. Because individuals are locked into cooperative partnerships, negative feedback enforces cooperation through the punishment of defection. The classic example of a partner-fidelity model is Trivers’ [4] theory of reciprocity in which individuals protect against exploitation by tracking another’s past behaviors and responding in kind (e.g., tit-for-tat), and thus cooperation is rewarded with cooperation, and free-riding is punished with defection [5]. Although an effective solution to the free rider problem, punishment is not the only way to maintain cooperation.

Another solution to the free-rider problem involves partner choice. Partner choice models allow individuals to freely select, for themselves, who they would like to interact with. Because individuals are free to choose their partners, the challenge becomes identifying – and being identified as – a cooperative individual. Given that past altruistic behaviors can be viewed as a signal of future willingness to cooperate, individuals with a history of altruism become more desirable social partners over time [6], [7]. Previous research suggests that these partner choice strategies can help maintain cooperation by effectively protecting populations from free-rider invasions [8] while encouraging altruistic behaviors [9], with minimal cognitive demands [10]. Indeed, it has been suggested that partner choice strategies, as compared to partner control, often have reduced cognitive constraints [10]–[12] and increased ecological validity (e.g., [3]).

Importantly, despite the substantial attention previously devoted to understanding the functions and mechanisms of partner control and partner choice strategies in adults (e.g., [3], [13]), we still know quite little about the early emergence of these behaviors in children. In particular, the extent to which early other-oriented behaviors are indiscriminate, only showing selectivity and specificity over the course of development, is presently a point of theoretical debate in the developmental literature [14]–[16]. There are a number of reasons to believe that even early in development children have the ability to make the evaluations necessary for effective partner choice in the domain of cooperative behavior. Specifically, past research has found that before their first birthday, infants prefer helpers over hinderers [17] and expect others to have similar preferences for helpers [18], likely foundations of partner choice. Further, by two years of age, children prefer to help individuals with positive intentions [19], and by 3-years, children utilize third party interactions to direct their helping behavior [20]–[22]. Taken together, the extant literature suggests that within the first two years of life human children possess the minimal abilities to engage in effective partner choice behavior. One important outstanding question regarding the emergence of partner choice behavior relates to how broadly children’s attributions of other’s partner qualities generalize.

Specifically, mature reciprocity goes well beyond trading the same good back and forth and instead requires the ability to evaluate and exchange diverse acts of comparable value [23]. To that end the present study is designed to determine the extent to which young children can be flexible in their identification of, and selective interaction with, good social partners. Communication is thought to play an important role in the evolution and maintenance of large-scale cooperation between unrelated and potentially novel individuals [24]–[26]. Through language, humans can access knowledge about intentions, past behavior, future plans, and a host of other information that would otherwise be inaccessible (e.g., [27], [28]). Indeed, the ability to engage in abstract, referential communication is thought to assist individuals in coordinating behaviors (e.g., [29]) and identifying other cooperators [30], [31]. Yet, the role of communication in the identification of, and selective interaction with, good social partners has not, to our knowledge, been directly tested in early development.

Although there is little work directly testing the relation between communication, social evaluation, and cooperative behavior, there are a number of reasons to believe that communication and cooperation are integrally entwined in humans. First, unlike other great ape species who only communicate to direct others’ behavior, humans often communicate for the sole purpose of sharing information and interest with others [32]. Relatedly, the tendency to spontaneously recognize and respond to communicative needs follows a developmental trajectory similar to that of responding to purely instrumental needs. Specifically, within the first two years of life children recognize when another individual is experiencing an instrumental challenge and readily offer help (e.g., by retrieving out of reach objects, or correcting unintended outcomes) [33]; similarly, by two years, children recognize when others are in need of information and readily provide that information through the use of pointing behavior [34]–[36]. Finally, significantly older children (5 years) who have evaluated an individual’s previous accuracy demonstrate a “halo” effect, predicting that in addition to being informative, the individual will also engage in other positive social behaviors [37].

In addition to their shared developmental trajectories, there is another reason to believe that communication may serve as a good test for the generalizability of children’s social evaluative abilities. Specifically, communication like cooperation allows individuals to gain benefits through interaction that would be unavailable to an individual acting alone, while simultaneously posing significant risks, if not exercised with caution. Indeed, individuals who are not selective about their communicative partners risk acquiring information that is wrong, mistaken, intentionally deceptive, or simply withheld (e.g., [38]). Previous research suggests that children’s acquisition of information from others’ communication shows selectivity and social evaluation similar to their evaluations of other’s cooperative behavior. For example, by 3 years, children can track an individual’s past reliability, preferentially seeking information (e.g., [39]) and learning [40] from individuals with a history of accuracy, even after a single interaction [41], suggesting that within the first four years of life children can track, evaluate, and selectively interact with others based on the quality of the information they have provided. The goal of the present study is to bridge two bodies of developmental literature and ask whether children can generalize their social evaluations and use the quality of an individual’s previous communicative behaviors to identify and selectively cooperate with good social partners. If communication is indeed viewed as a cooperative act, and if children can generalize their social evaluations across diverse behaviors, three predictions follow: 1) children should explicitly identify informative individuals as helpful (Experiments 1 and 2); 2) children should choose to share information with partners who have previously been informative (Experiment 1); and 3) children should selectively reciprocate informational acts with other forms of cooperation (specifically, instrumental helping; Experiment 2).

## Experiment 1

In Experiment 1 we examined two related questions. First, do children explicitly identify individuals who share accurate information as helpful? Second, do children selectively provide helpful information to previously informative individuals?

### Method

#### Participants

Twenty-nine 3-year-old children (M = 40.79 months, 15 female) participated in the study. Five additional children were excluded from analysis due to experimenter error (*n = *3), parental interference (*n = *1), and language delays (*n = *1). The Queen's University Committee on the General Research Ethics Board approved the ethics of this study. Informed consent, in written form, was obtained from the parents of all children who participated in this study.

#### Procedure

Participants were brought into the testing room by a female experimenter (E1) and situated across a low table from two small monkey puppets. A second female experimenter (E2) operated both of the puppets to ensure consistency and reduce bias. Parents were seated behind the children and were asked not to interact with their children. During the familiarization phase, E1 introduced the children to the puppets and encouraged the children to greet them. Puppets were chosen because previous research suggests that children readily interact with puppets as social entities (e.g., [42]).

After the children were introduced to both puppets, E1 informed the children that their task was to identify four pictures. The pictures were of common, familiar objects (apple, t-shirt, cupcake, dog) hidden behind a yellow mask, revealing only a small, uninformative section of the image. The children were then encouraged to ask the puppets about the identity of the picture. To ensure appropriate control and counterbalancing, E1 directed the questioning. In turn, each puppet would advance, look down at the picture and then back at the child, and provide a scripted response that varied across puppet. One of the puppets was *informative* whereas the other was *withholding* to inform. The accurate informer responded with “I know! It’s an (accurate item)”, always providing a noun that correctly identified the hidden picture. In contrast, the withholding informer would respond with “I know! But I’m not telling”, in a friendly yet straightforward manner. After both puppets had provided a response, E1 would remove the mask to reveal the hidden picture. The experimenter would then confirm that the child knew what the picture was before setting up the next picture on the table. The same procedure was repeated for four pictures. The location, order, and shirt color (red and blue), of the informative versus withholding puppet was counterbalanced across participants.

Following the four familiarization trials, E1 directed the child’s attention to another picture that was hidden face-down on the floor. The experimenter explained that the puppets had never seen this new picture before and invited the children to “take a peek” at the picture with her. After showing the children the new picture, the experimenter replaced the mask and placed the masked picture on the table in front of the puppets. E2 then advanced both puppets in unison towards the picture. The puppets looked down at the photo, back at the child, and then said “Hmm”. They gazed alternately a second time and then vocalized in unison “I wonder what that is?”. The children were then given an opportunity to provide the puppets with information regarding the identity of the picture. Pilot testing revealed that spontaneous informing after the puppets displayed interest was rare. Therefore, E1 provided the children with a prompt: “Would you like to help one of the puppets? Which puppet would you like to help?”. The prompt served two functions: (1) it established the child’s ability to reveal the identity of the picture (they had been cued to be quiet when they were first looking at the picture), and (2) the prompt helped to minimize vague responses. Previous selective helping tasks have utilized an object retrieval paradigm where there was a single item that could be returned to a single individual. Information, unlike objects, is not inherently bounded and thus it was possible for children to reveal the information to both puppets at once. The use of the cue encouraged selectivity, without explicitly telling the child how to help.

Informing behavior consisted of approaching one of the puppets and informing it what was hidden behind the mask. Children could inform in two ways: they could show (by removing the mask) or tell the puppet the identity of the hidden picture. If a child made no response, the experimenter would end the helping trial by removing the covered picture from the table.

Once the picture had been removed, the children were asked to identify 1) the puppet that they thought was helpful and 2) the one that they thought was sneaky. Children’s responses were coded based on their pointing behavior. These questions allowed us to ensure that the children remembered the manipulation, explicitly viewed information sharing as prosocial and, finally, by asking about the sneaky puppet we could ensure that the children were not simply adverse to approaching the withholding puppet.

An experimenter blind to the research hypotheses re-coded all of the participant’s behavior via video recording (N = 22); interrater reliability was high (Agreement: Helping 100%, Helpful, 95%, Sneaky 95%).

### Results and Discussion

Twenty-two of the twenty-nine children (75.86%) clearly provided information to a single puppet. Of the seven remaining children, five declined to help either of the puppets and two identified a single puppet to help, but then failed to provide them with information. (Both of these children selected the informative puppet as the helping recipient. Further, they both identified the informative puppet as helpful, and the withholding puppet as sneaky). All seven were excluded from further analysis. Six of the twenty-two helpers verbally told one of the puppets the identity of the hidden picture (27.27%), fifteen showed the puppet by holding up the picture (68.18%), and one child did both (4.5%).

**Figure 1:**
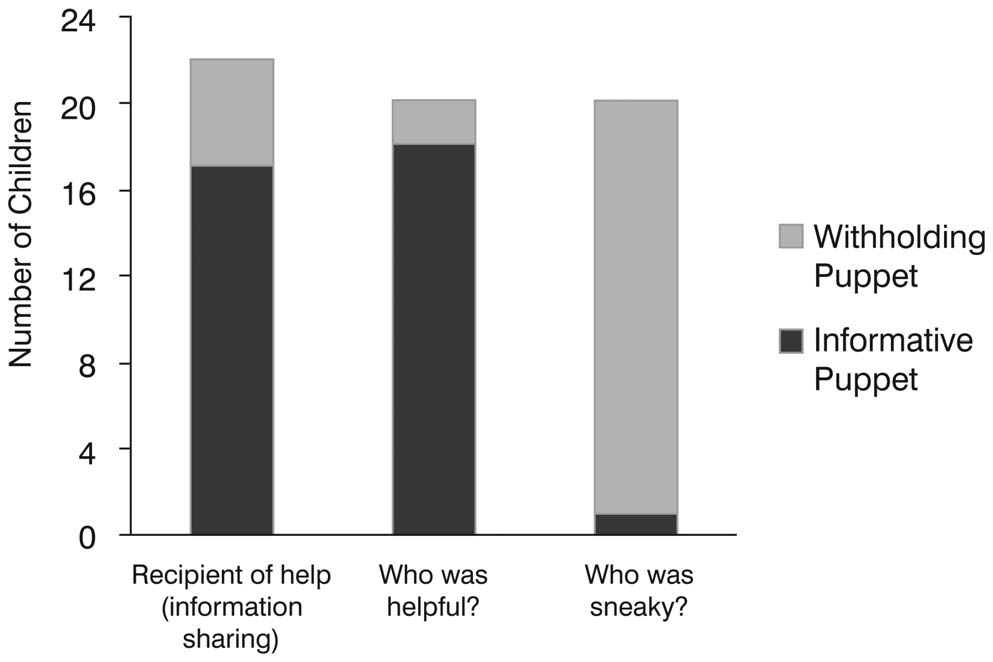
Results of Experiment 1 showing the number of children choosing the accurate versus the withholding puppet across the three types of test trials. All binomial comparisons are significant at *p*<.02.

If the children evaluated the puppets’ past behavior and selected their cooperative partner based on the partner’s expressed willingness to help, then they would have shown a preference for sharing information with the informative puppet. Consistent with this proposal, there was a significant preference for helping the informative puppet (n = 17, 77.3%) over the withholding puppet (n = 5, 22.7%; binomial analysis, *p = *.02; Figure 1). Moreover, when asked to identify the “helpful” puppet, children overwhelming endorsed the informative puppet (n = 18) as opposed to the withholding puppet (n = 2, binomial analysis, *p* = .006; Figure 1). Importantly, when asked to identify the “sneaky” puppet, children showed the opposite pattern, identifying the withholding puppet (n = 19) as opposed to the informative puppet (n = 1, binomial analysis, *p = *.001; Figure 1). Two children identified both puppets as helpful *and* sneaky and were therefore excluded from the analysis.

Taken together, this pattern of responses suggest that in addition to explicitly identifying informative communication as a helpful act, children can also utilize their understanding of communicative intent to identify good social partners. These findings complement the literature on selective information seeking by demonstrating that children are not only selective in their consumption of information (e.g., [39], [40]) but also in their provision of information. Moreover, the observed pattern of selectively communicating with previously informative individuals and endorsing informative individuals as “helpful” is consistent with the hypothesis that communication serves an important role in monitoring and maintaining cooperation (e.g., [24]–[26]). On the basis of these results, we used a similar information-sharing paradigm to test whether children utilize a partner’s communicative tendencies to direct their partner choice behavior in a different domain of cooperation, namely instrumental helping.

## Experiment 2

Experiment 1 demonstrated that children evaluate individuals who willingly communicate as better social partners and preferentially share information with previously informative individuals. However, selective prosociality does not always involve trading the same item or act back and forth, but instead requires the ability generalizing across diverse displays of cooperation [23]. Thus, a stronger test of the flexibility of children’s early partner choice behaviors involves examining their ability to respond to a partner’s display of cooperative intent with a distinct cooperative act. To this end, Experiment 2 was designed to test whether children use an informant’s past communicative behavior to direct another form of cooperation, namely instrumental helping. Specifically, we asked whether three-year-old children (n = 27) could use information about one type of cooperative behavior (information sharing) to identify a good social partner and then selectively reciprocate with a distinct variety of cooperation (retrieving out-of-reach objects).

### Methods

#### Participants

Twenty-seven 3-year-old children (M = 42.57 months, 14 female) participated in Experiment 2. Eight additional children were excluded from analysis due to experimenter error (*n = *3), parental interference (*n = *3), failure to interact with the puppets (*n = *1), and no video recording (*n = *1).

#### Procedure

Experiment 2 utilized the same familiarization procedure as Experiment 1. However, instead of giving the children an opportunity to share information with the puppets, the children were given an opportunity to engage in instrumental helping. Following familiarization, the two puppets were offered a toy, which subsequently fell out of their reach onto the floor [33]. Both of the puppets then reached over the edge of the table in an attempt to retrieve it, thus providing children with an opportunity to help (19). Unlike previous studies using a similar methodology, the children in this study had not previously traded any items with the experimenter or the puppets, and thus, children who did not spontaneously and immediately help (by retrieving the toy) or who engaged in unclear choices (because their eyes and arms were not directed towards the same target, or because their offer was directed towards the experimenter operating the puppet) were asked, “Could you help one of the puppets?”. After the helping task, two questions examined children’s evaluation of the puppets’ previous behavior: children were shown a new masked picture and asked to select the puppet that they thought would be able to help identify the image, and children were asked to identify the “helpful” puppet. A blind coder coded all of the videos (N = 24) to establish interrater reliability; interrater reliability was high (Agreement: Helping 94%, Asking, 100%, Helpful 100%).

### Results and Discussion

Twenty-four of the 27 (88.89%) children helped a puppet by retrieving the out-of-reach object. Seven of these children spontaneously helped one of the puppets (29.17%), six children spontaneously retrieved the object but were questioned to clarify the recipient (25%), five children retrieve the dropped object after being asked if they could help but then spontaneously selected a single recipient (20.83%), and six children were asked twice if they wanted to help one of the puppets (once to retrieve the toy, and once to specify the recipient; 25%). Unlike Experiment 1, no children selected a target but then failed to help.

**Figure 2:**
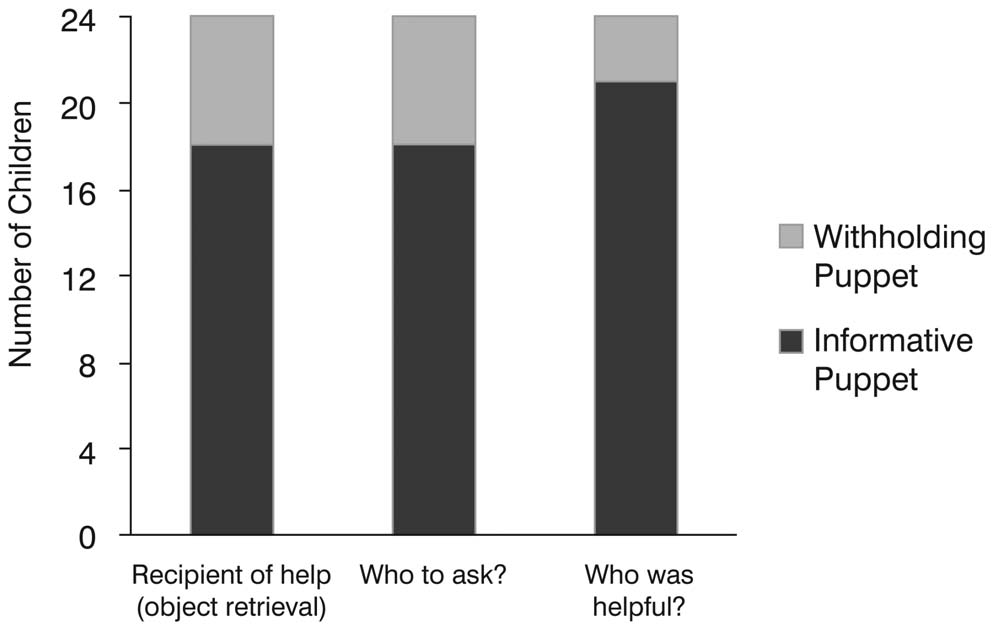
Results of Experiment 2 showing the number of children choosing the accurate versus the withholding puppet across the three types of test trials. All binomial comparisons are significant at *p*<.02.

Consistent with an ability to generalize across diverse cooperative acts in the identification and selection of good social partners, and consistent with a functional relation between communication and cooperation, children preferentially helped the informative (n = 18) as opposed to the withholding puppet (n = 6, binomial analysis, *p*<.02; Figure 2) by retrieving out-of-reach objects. Moreover, children used their evaluation of the puppet’s previous communication to preferentially direct new questions back to the informative (n = 18) as opposed to the withholding puppet (n = 6, binomial analysis *p*<.02; Figure 2), suggesting that the children remembered not only who was more deserving of help, but also why. Finally, replicating the results of Experiment 1, the children explicitly identified the informative (n = 21) as opposed to the withholding puppet (n = 3) as helpful (binomial analysis, *p = *.002; Figure 2).

The results of Experiment 2 demonstrate that early cooperation is both selective in terms of recipient and flexible in terms of specific cooperative act. Moreover, it provides evidence that the ability to monitor and evaluate communicative quality influences the tendency to engage in a diverse suite of cooperative behaviors, suggesting that social evaluations are formed with equal facility based on both communication and cooperation. Specifically, from early in development, children can utilize social evaluations from a number of different acts to identify good social partners and explicitly identify communicative individuals as ‘helpful’ and generalizing cooperative behaviors across diverse contexts (i.e., information sharing and retrieving out of reach objects).

## General Discussion

Many have argued for species-specific cognitive and motivational abilities that underlie the ubiquitous human tendency to cooperate [11], [12], [43], [44]. The shared ability to identify, and preferentially interact with other cooperators through partner choice behaviors is also thought to be integral to the complexity of human cooperative interactions [3]. Selective partner choice works as a protective mechanism against both free riding and deception because individuals can utilize past behavior to inform decisions regarding subsequent social interactions. To that end, children’s preference to communicate (Experiment 1) and cooperate (Experiment 2) with the communicative individual, while explicitly identifying communicative individuals as cooperative (Experiments 1 & 2), suggests that children can flexibly generalize their identification of, and selective interactions with, good social partners across diverse acts.

Importantly, the ease with which the children utilized their evaluations of an individual’s communicative intent to select a good social partner is especially compelling support for the fundamental relation between communication and cooperation because these findings are consistent with past research demonstrating that children are particularly good at predicting consistency in cooperative behavior [45] even when they are displaying difficulty making behavioral predictions in other domains [46]–[48]. Moreover, the children in our study utilized their observations of past communicative behavior to direct their selection of a cooperative partner, even in the absence of explicit reference to the potential utility of the observations during the puzzle task (see [41], [45]).

Although these studies demonstrate that by 3-years children have the capacity to use past communication to identify and selectively interact with cooperators, it is possible that there are situational constraints on the spontaneous use of this strategy. By limiting the children’s resources, we created a situation in which they were required to be choosy cooperators. Indeed, given children’s proclivity towards helping others based on minimal past interactions (e.g., [33], [49]), it is possible (if not probable) that when resources are abundant, and helping involves little cost, children will be less inclined to show such a strong partner bias (e.g., [50]). Yet, we urge caution in considering this as a limitation of the design because selecting between two or more potential partners in situations of scant resources *is* the basis of partner choice strategies (e.g., [2], [10]).

The demonstration of children’s ability to utilize communicative intent to identify good cooperators opens the door to a number of directions for future research. First, testing the limits of early partner choice, and the specific nature of the relation between communication and cooperation, requires a movement beyond the domain of helping behavior. Human cooperative interactions are diverse (e.g., [51]–[53]). People can respond to other’s displays of instrumental need with help, their material desires with sharing, and their emotional distress with comfort [51]. Each of these behaviors is thought to rely on distinct social-cognitive skills, and shows unique developmental trajectories [54]. To that end, if the ability to identify and selectively interact with cooperative individuals is a fundamental mechanism that supports cooperation, and communication is a variety of cooperation, the association should be observable in other domains of human cooperation.

### Conclusion

In sum, the present study provides some of the first evidence that communication and cooperation are integrated in early development. Specifically, we demonstrate that children evaluate communicative acts as cooperative, utilize communicative intent to identify and selectively aid good social partners, and reciprocate across diverse displays of cooperation. Taken together, these findings bolster claims regarding the integral role that communication plays in the maintenance of human cooperative behavior and supports the suggestion that even early in development, humans are predisposed to appropriately – and selectively – exploit the potential benefits of cooperation through the use of appropriate partner choice behaviors.
